# Trends in gestational age and birth weight in Chile, 1991–2008. A descriptive epidemiological study

**DOI:** 10.1186/1471-2393-12-121

**Published:** 2012-11-02

**Authors:** Paulina O Lopez, Gérard Bréart

**Affiliations:** 1 INSERM, UMR S953, Recherche Epidémiologique en santé périnatale et santé des femmes et des enfants, Hôpital Tenon, Paris, F-75020, France; 2 Université Pierre et Marie Curie, UMR S 953, Paris, F-75005, France; 3 Universidad de Valparaíso Escuela de Obstetricia y Puericultura, Blas Cuevas 1028, Valparaíso, Chile

## Abstract

**Background:**

Gestational age and birth weight are the principal determinants of newborn’s health status. Chile, a middle income country traditionally has public policies that promote maternal and child health. The availability of an exhaustive database of live births has allows us to monitor over time indicators of newborns health.

**Methods:**

This descriptive epidemiological study included all live births in Chile, both singleton and multiple, from 1991 through 2008. Trends in gestational age affected the rate of prevalence (%) of preterm births (<37 weeks, including the categories < 32 and 32–36 weeks), term births (37–41) and postterm births (42 weeks or more). Trends in birth weight affected the prevalence of births < 1500 g, 1500–2499 g, 2500–3999 g, and 4000 g or more.

**Results:**

Data from an exhaustive register of live births showed that the number of term and postterm births decreased and the number of multiple births increased significantly. Birth weights exceeding 4000 g did not vary.

Total preterm births rose from 5.0% to 6.6%, with increases of 28% for the singletons and 31% for multiple births (p for trend < 0.0001). Some categories increased even more: specifically preterm birth < 32 weeks increased 32.3% for singletons and 50.6% for multiple births (p for trend 0.0001).

The overall rate of low birth weight infants (<2500 g) increased from 4.6% to 5.3%. This variation was not statistically significant for singletons (p for trend = 0.06), but specific analyses exhibited an important increase in the category weighing <1500 g (42%) similar to that observed in multiple births (43%).

**Conclusions:**

The gestational age and birth weight of live born child have significantly changed over the past two decades in Chile. Monitoring only overall rates of preterm births and low-birth-weight could provide restricted information of this important problem to public health. Monitoring them by specific categories provides a solid basis for planning interventions to reduce adverse perinatal outcomes.

This epidemiological information also showed the need to assess several factors that could contribute to explain these trends, as the demographics changes, medical interventions and the increasing probability of survival of extremely and very preterm child.

## Background

Gestational age (GA) and birth weight (BW) are the principal determinants of health status at birth because they are closely linked to neonatal survival, neonatal and infant morbidity, and later, in adults, to potential sequelae and quality of life [1-4]. The World Health Organisation (WHO) recommends that these two measures be recorded to assess and monitor perinatal results linked to the duration of pregnancy and the principal health conditions at birth; such records would help to guide policies related to mothers’ and children’s health [5]. Otherwise, the distribution of GA and BW according to vital status and according to the number of infants born is an essential outcome indicator in the EURO-PERISTAT project for monitoring and assessing perinatal health in 26 European countries [6].

A series of reports from the International Conference on Prematurity and Stillbirths (USA 2009) reports the damage caused by preterm birth in the world and shows that it is a public health problem on a global scale currently increasing in most countries [1]. The Conference recommends that preterm births must be measured more accurately with data of high quality and with standard definitions; particularly specific and overall rates should be analyzed according to internationally comparable categories [1,4].

Currently, one of the most important issues is the lack of knowledge about the magnitude and impact of PTB in the world. This barrier is also related to the lack of visibility of this problem. One of the explanations to this barrier is the unavailability of data concerning vital statistics, because births are not always routinely registered [7].

In Chile, preterm birth became an important health priority during the recently enacted health reform, because it is the principal cause of perinatal morbidity and mortality as well as of admission to neonatal intensive care units. Premature newborns weighing <1500 g account for approximately 50% to 70% of neonatal mortality and then 25% to 30% of infant mortality [7].

Preterm birth is the cause of the short- and long-term sequelae of varying severity that affect 23% of births before 32 weeks. Chilean researches showed that after two years of follow-up, 18% of these children have permanent disabilities, still of variable severity. These can be distributed as follows: 5% disabling, 8% language impairment, and 5% other (diverse sequelae) [7,8].

Moreover, the economic cost of a premature baby, which are borne by the families and the health-care system, is enormous for a country such as Chile, with a medium income level, with a mixed (public and private) health care system and large socioeconomic disparities [3]. An study made in one of the most important hospital in Santiago, showed that the total mean cost of cares for each preterm newborn weighing less than 1500 grams was 12 017 650 CLP (21 467 USD) in 2004 and could go as high as CLP 43 932 072 (USD 78 474). This cost includes hospital services but not includes surgeries. The principal cost is the occupation of the hospital bed (around 65% of total cost). In this study, the average length for hospital stay for preterm child with 32 weeks of GA was 34 days and could go until 100 days at 26–27 weeks of GA [9].

Our objective is to analyse the trends in GA and BW in the population of live births over the past two decades. More particularly, these analyses are intended to measure not only the trends in overall prevalence but also the specific prevalence of both preterm birth and LBW according to different categories.

These categories present important differences in terms of neonatal mortality, morbidity and also the need of financial resources [1].

## Methods

This descriptive epidemiologic study included the general population of all live births in Chile (N = 4 559 917) from 1991 through 2008 as recorded in the National Database of Live Births.

### Chilean database and register of live births

This database was established by an agreement in 1982 between the Civil Registry of Chile, the National Institute of Statistics (INE) and the Ministry of Health (MINSAL) as part of the process of computerization of vital statistics, is the official source for all maternal and perinatal statistics as well as health indicators for live births. It records live births including home births. Stillbirths are not included.

The database is routinely validated by comparison with hospital archives before being available for statistical or research purposes. The access is opened to persons who apply and justify its use. A password is thus provides [10].

In Chile, prenatal care has a universal access. Consequently, 99.8% of births take place in health care facilities, with healthcare professionals [11]. The information is thus collected by obstetricians and midwives in a delivery certificate. This certificate serves as the link between the maternity ward and the vital records office where births are recorded.

The register’s criterion of live birth is that recommended by the WHO: a GA of at least 22 completed weeks or a birth weight of at least 500 g [12].

### Gestational age measures

Gestational age was estimated by the physician or midwife according to the WHO recommendations from the date of the first day of the last menstrual period (LMP) at the beginning of prenatal care. Date was confirmed later from ultrasound (USN) during the first trimester [13]. BW was also measured according to WHO recommendations [14].

During the 18 year period, the use of ultrasound to asses GA increased progressively. During the 1991–1995 period (P1), the proportion of USN confirmation was around 60%; from 1996 through 200 (P2) was more commonly used (around 75%) and performed in all cases with uncertain LMP dates or with discordant obstetric examinations. During the third period (P3), according to National guidelines USN became routine and replaced the estimate based on the date of the last menstrual period if it differed from the later by more than seven days [15].

In order to observe the possible influence of increasing use of USN on the results, the analysis was conducted along these 3 periods.

### Exclusion criteria

In order to obtain reliable classifications, this study excluded births with a birth weight less than 500 g or a gestational age less than 22 weeks, missing GA values, weight, or length at birth, gestational age values ≥ 44 weeks, and classification errors for gestational age (also referred to as misclassifications).

Misclassifications of GA were identified by their outlying values of weight and length at birth. We used Tukey’s statistical rules, as applied by Arbuckle in Canada [16]; she treated as outliers the measurements located at a distance equal to or greater than 1.5 times the interquartile range.

These reference limits were obtained from the birth weight and length distribution in the population and applied by week of GA to the distribution from 22 to 43 weeks. This method was chosen after a graphic examination of the outliers and the effect of their elimination on the final distribution.

### Main outcomes and steps in the analysis

The first objective of the analysis was to examine the register and the GA and BW measurements by looking at the trends for the excluded values. Next we looked at the effect of the exclusions on the principal results: prevalence rates of preterm births and LBW.

We then analysed the trends in the distribution of GA in completed weeks, as follows:

22–36 weeks (preterm birth).

37–41 weeks (term birth).

≥ 42 weeks (postterm birth).

The preterm births were subdivided into two groups: <32 weeks (including extremely and very preterm births) and 32–36 weeks (moderately preterm births) [4].

Trends in the distribution of BW in grams were analysed in four categories: < 1500 g and 1500–2499 g, 2500–3999 g, ≥4000 g.

The trends in each category of GA and BW from one period to another were measured by the proportion of each category among all births (%) and the 95% confidence interval of each. This percentage corresponds to prevalence rates per 100 live births and by period in the general population. To compare two periods, we used the percentage of change or relative variation: ([(% final period -% initial period)/% initial period] *100). To examine the specific trends, the analyses were performed for all births and separately for singletons and multiple births (with more than one newborn). Analyses of overall trends were performed with Prais-Winsten regression models for times series by year. In this model, the errors are assumed by a first-order autoregressive process. Differences of rates between periods were evaluated by Pearson’s Chi square test.

The data management and statistical analyses were performed with STATA SE; Statistics Data Analysis software, version 10.0.

## Results

### Birth registration in the national database

Table 1 shows the recording of measures excluded of GA and BW and allow us to see the relatively low frequencies for each type of excluded value throughout the three periods and the progressive reduction of errors and in missing values for GA and for BW.

**Table 1:** Values excluded from the Chilean National Database of Live Births, by year

**Periods**	**Total births**	**GA**^**a**^ **< 22**	**%**	**BW**^**b**^ **< 500**	**%**	**Errors**	**%**	**GA missing**	**%**	**GA** ≥44	**%**	**BW missing**	**%**
						**values**							
1991-1995	1 379 194	124	0.01	211	0.02	29 205	2.12	5 471	0.40	164	0.01	4441	0.32
1996-2000	1 281 424	99	0.01	198	0.02	25 104	1.96	2 799	0.22	111	0.01	2006	0.16
2001-2008	1 899 299	525	0.03	960	0.05	24 720	1.3	2 482	0.13	544	0.03	2310	0.12
Total	4 559 917	748	0.02	1369	0.03	79 029	1.73	10 752	0.23	819	0.02	8757	0.19

^a^Gestational age.

^b^Birth weight.

The overall frequency of records not meeting the definitions that is: births with GA < 22 and/or LBW < 500 g, was very low and concerned only 1584 observations (0.035%), but their number increased over time. Among them, 84% were found at 20 or 21 weeks of GA, between 1991 and 2001; this subgroup subsequently fell to 77%. The trend in GA values ≥ 44 clearly decreased through 2000; the analysis by year (data not shown) indicates that they peaked in 2003 and 2004 (n = 543), but after 2004 reached a frequency = 0. Overall, 92 184 (2%) observations were removed from the initial set, and the population serving as the basis for classification was 4 467 733 births.

**Figure 1:**
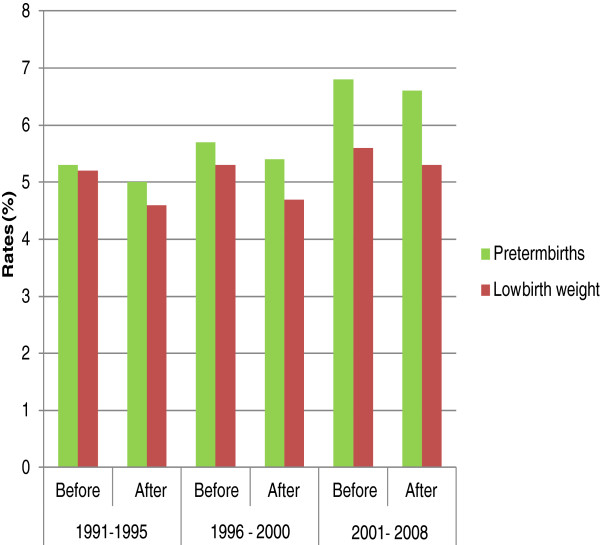
Rates of PTB and LBW before and after exclusions in Chilean database of live births.

This proportion of excluded values did not modify the trends for the prevalence of preterm birth or LBW (Figure 1).

**Figure 2:**
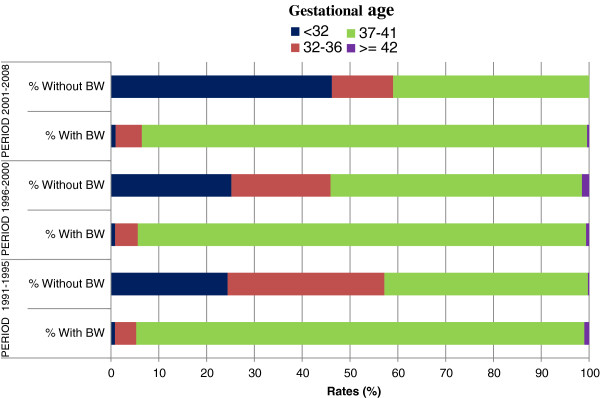
**Distribution of GA**^**a**^**by availability of BW**^**b**^**measurement in Chilean data base of live births.**

**Figure 3:**
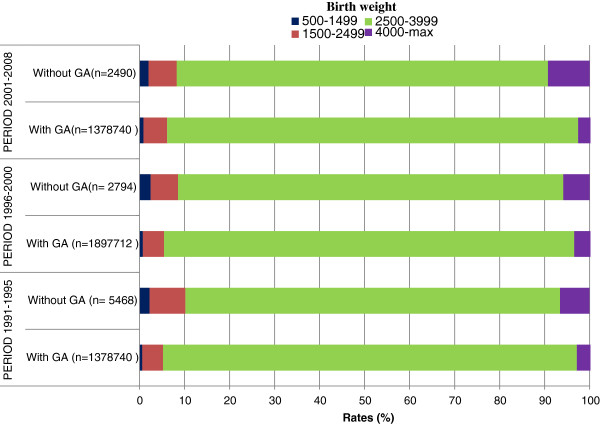
**Distribution of BW**^**a**^**by availability of GA**^**b**^**measurement in Chilean data base of live births.**

Despite the very low number, missing values are a specific subgroup and was analyzed separately. In fact, the LBW rate for births with missing GA values was higher than that of the general population (Figure 2), and those with missing BW values had a very high rate of PTB (Figure 3).

### Trends in gestational ages and low birth weights

Table 2 shows the trends in the distributions of gestational age and their confidence intervals, as well as the statistical importance of the variations over time. We can observe in the overall population (singleton and multiple births), that all births < 37 weeks of gestation increased in 32% (ranging from 5.0% in 1991 to 6.6% in 2008). This trend was accompanied by a reduction in term births, which began in 1996 for singletons but was observed from the beginning of the period among multiple pregnancies. A large reduction in postterm births was observed among singletons although in multiple births these rates did not vary significantly over the entire period. Beyond the overall preterm birth rate, the increase in preterm births concerned principally the category born before 32 weeks during all three periods.

**Table 2:** Trends of gestational-age categories, singletons and multiples live births in Chile 1991-2008

**Period**	**1991-1995**	**1996-2000**	**2001-2008**	**P1**^**a**^	**P2**	**P3**	**P1P2**		**P2P3**		**P1P3**		**Overall Trend**
**Total population**	1 344 067	1 253 164	1 870 502										
4 467 733													
Gestational age^e^	Rate %				95% CI^b^		% change	*P*^*c*^	% change	*P*	% change	*P*	*P*^*d*^
<32	0.70	0.77	0.96	[0.68-0.71]	[0.76-0.79]	[0.95-0.98]	10.0	*< 0.000*	24.7	*< 0.000*	37.1	*< 0.000*	*< 0.000*
32-36	4.33	4.64	5.62	[4.30-4.37]	[4.60-4.68]	[5.59-5.66]	7.2	*< 0.000*	21.1	*< 0.000*	29.8	*< 0.000*	*< 0.000*
37-41	93.98	93.96	93.05	[93.9-94.02]	[93.9-94.4.00]	[93.0-93.10]	0.0	*0.47*	−1.0	*< 0.000*	−1.0	*< 0.000*	*0.004*
≥42	0.99	0.62	0.36	[0.97-1.00]	[0.61-0.63]	[0.35-0.37]	−37.4	*< 0.000*	−41.9	*< 0.000*	−63.6	*< 0.000*	*< 0.000*
**Singletons**	(1 323 021)	(1 232 824)	(1 836 582)										
(4 392 427)													
<32	0.62	0.67	0.82	[0.60-0.63]	[0.66-0.69]	[0.81-0.84]	8.1	*< 0.000*	22.4	*< 0.000*	32.3	*< 0.000*	*< 0.000*
32-36	3.80	4.04	4.83	[3.76-3.83]	[4.00-4.07]	[4.80-4.86]	6.3	*< 0.000*	19.6	*< 0.000*	27.1	*< 0.000*	*< 0.000*
37-41	94.58	94.66	93.98	[94.5-94.62]	[94.6-94.70]	[93.9-94.01]	0.0	*0.48*	−0.7	*< 0.000*	−0.6	*< 0.000*	*0.020*
≥42	0.99	0.63	0.37	[0.98-1.00]	[0.62-0.64]	[0.36-0.38]	−37.0	*< 0.000*	−41.3	*< 0.000*	−63.0	*< 0.000*	*< 0.000*
**Multiples (**75 306)	(21 046)	(20 340)	(33 920)										
<32	5.63	6.84	8.48	[5.30-5.90]	[6.50-7.20]	[8.20-8.80]	21.5	*<0.01*	24.0	*< 0.000*	50.6	*< 0.000*	*< 0.000*
32-36	38.00	41.50	48.60	[37.4-38.80]	[40.81-42.17]	[48.04-49.10]	9.2	*<0.01*	17.1	*< 0.000*	27.9	*< 0.000*	*< 0.000*
37-41	55.80	51.65	42.93	[55.6-56.90]	[51.00-52.34]	[42.41-43.46]	−7.5	*<0.01*	−16.9	*< 0.000*	−30.0	*< 0.000*	*< 0.000*
≥42	0.04	0.01	0.01	[0.02-0.07]	[0.00-0.02]	[0.00-0.02]	−75.0	*0.012*	0.0	*0.629*	−75.0	*< 0.000*	*0.623*

^a^Periods: P1: 1991–1995, P2: 1996–2000, P3:2001–2008.

^b^95% confidence interval.

^c^Pearson’s chi-square test with significance level for p < 0.05.

^d^Prais-Winsten Regression analysis for trend with significance level for p < 0.05.

^e^Gestational age in weeks.

In multiple births, the rates of preterm and LBW throughout the three periods were 10 times higher than those of singleton births.

Multiple births increased significantly: 1.57% in P1 [95% CI: 1.54, 1.58], 1.62% in P2 [95% CI: 1.60, 1.64], and 1.81% in P3 [95% CI: 1.80, 1.83]. The increase involved mainly twin births, because the triplet rate since 2001 has remained stable, around 0.04%, and the quadruplet rate was 0% (data not shown).

Table 3 shows the increase in births with less than 2500 g. We can observe in the overall population (singleton and multiple births), that all births with less than 2500 g increased in 13.8% (from 4.62% in 1991 to 5.27% in 2008). This increase is significant among multiple births (*p* for trend < 0.0001) but not among singletons (*p* for trend = 0.06). This difference may be explained by different trends for the 1500-2499-g category, which fell among singletons until 1996 and increased thereafter, while among multiple births, this category increased during three periods. Nonetheless, the category of babies weighing <1500 g increased substantially both among singletons and multiple births and at a similar rate in both groups. Finally, the proportion of infants in the weight category of 2500–3999 g fell among both singleton and multiple births.

**Table 3:** Trends of birth weight categories, singletons and multiples live births in Chile 1991-2008

**Period**	**1991-1995**	**1996-2000**	**2001-2008**	**P1**^**a**^	**P2**	**P3**	**P1P2**		**P2P3**		**P1P3**		**Overall Trend**
**Total population**	1 344 067	1 253 164	1 870 502										
N = 4 467 733													
Birth weight^e^	Rate %				95% CI^b^		% change	*P*^*c*^	% change	*P*	% change	*P*	*P*^*d*^
<1500	0.61	0.72	0.88	[0.60-0.62]	[0.70-0.73]	[0.87-0.90]	18	*< 0.000*	22.2	*< 0.000*	44.3	*< 0.000*	< 0.000
1500-2499	4.02	3.99	4.39	[4.00-4.05]	[3.95-4.02]	[4.36-4.41]	−0.7	*< 0.000*	10.0	*< 0.000*	9.2	*< 0.000*	0.126
2500-3999	87.27	86.43	86.01	[87.22-87.33]	[86.37-86.49]	[85.96-86.06]	−1.0	*< 0.000*	−0.5	*< 0.000*	−1.4	*< 0.000*	< 0.000
4000-max	8.10	8.86	8.72	[8.05-8.15]	[8.80-8.90]	[8.68-8.77]	9.4	*< 0.000*	−1.6	*< 0.000*	7.7	*0.00*	0.190
**Singletons**	(1 323 021)	(1 232 824)	(1 836 582)										
(4 392427)													
<1500	0.53	0.62	0.75	[0.52-0.54]	[0.60-0.63]	[0.74-0.76]	17.0	*< 0.000*	21.0	*< 0.000*	41.5	*< 0.000*	< 0.000
1500-2499	3.44	3.38	3.65	[3.42-3.48]	[3.35-3.41]	[3.60-3.70]	−1.7	*< 0.000*	8.0	*< 0.000*	6.1	*< 0.000*	0.274
2500-3999	87.80	87.00	86.70	[87.74-87.85]	[86.94-87.06]	[86.64-86.77]	−0.9	*0.000*	−0.3	*< 0.000*	−1.3	*< 0.000*	0.003
4000-max	8.23	9.00	8.90	[8.17-8.27]	[8.95-9.05]	[8.83-8.92]	9.4	*< 0.000*	−1.1	*< 0.000*	8.1	*0.000*	0.176
**Multiples (**75 306)	(21 046)	(20 340)	(33 920)										
<1500	5.53	6.70	7.89	[5.20-5.80]	[6.40-6.90]	[7.60-8.20]	21.2	*< 0.000*	17.8	*< 0.000*	42.7	*< 0.000*	< 0.000
1500-2499	39.87	40.77	44.43	[39.20-40.53]	[40.09-41.44]	[43.90-45.02]	2.3	*0.01*	9.0	*< 0.000*	11.4	*< 0.000*	0.006
2500-3999	54.38	52.37	47.52	[53.71-55.06]	[51.69-53.06]	[47.00-48.05]	−3.7	*< 0.000*	−9.3	*< 0.000*	−12.6	*< 0.000*	< 0.001
4000-max	0.22	0.16	0.15	[0.16-0.28]	[0.10-0.21]	[0.11-0.19]	−27.3	*< 0.000*	−6.3	*< 0.000*	−31.8	*<0.000*	0.560

^a^Periods: P1: 1991–1995, P2: 1996–2000, P3:2001–2008.

^b^95% confidence interval.

^c^Pearson’s chi-square test with significance level for p < 0.05.

^d^Prais-Winsten Regression analysis for trend with significance level for p < 0.05.

^e^Birth weight in grams.

Although overall trend of the category of birth weights greater than 4000 g remained stable as a whole for all births, the trend by type of birth was also different; since this group increased in singletons between the first and second period and fell after 2001, while among multiple births, this group declined through the three periods.

Apparently, there were two types of trends, those for singletons, which were more recent, and those for multiple births, which were present before the observation period.

## Discussion

In the overall population, the trends in gestational age and BW show a significant increase in preterm birth and low BW, a reduction in term and postterm births and a stable level of birth weights above 4000 grams.

Preterm births increased most among births before 32 weeks, and LBW births in the category with BW < 1500 g. These trends deserve special attention because these groups of live births have higher known risks of mortality, morbidity, and sequelae [7,17,18], and also because these trends were most pronounced during the most recent period. Another trend to consider is the increase in multiple births because this group continues to have a high prevalence of prematurity, exceeding 50% during the last period

These trends seem to correspond to a real change; they have been obtained from a database where the records of the variables of interest can be considered good and its exhaustiveness has been observed through the three periods, expressed by a very low proportion of missing values. The observed changes were significant and consistent across all three periods, they involved the entire population of births and affecting singletons and multiple births. The measurements recorded are also highly biologically plausible, in terms of the very small proportion of misclassifications and values ≥44 weeks. Similarly, the trend in birth weight was consistent with that of gestational age.

Although the aim of this study is descriptive and not explanatory, the principal limitation of our research, like all research from general population databases, was the lack of more precise information to enable us to answer more specific questions [19].

Therefore, we lack more precise information of the method used to asses individual GA. Thus we cannot differentiate between those cases evaluated with one method or the other nor those GA corrected by USN and those not.

We miss as well an association with the Stillbirth registry, which prevented us from obtaining complete information about changes in GA and BW for those who die before birth.

Despite such limitations, it appears useful to focus the discussion on the possible effects on these trends of changes in obstetric and perinatal management that are capable of modifying the distribution of births at the population scale to shorten the duration of gestation. This focus should help to guide the formulation of basic hypotheses for future research.

### The potential influence of some factors that could modify the distribution of births

The World Health Organization explains that the increase in register of live birth is associated to the possibilities of survival where the birth took place [20]. Then we may reasonably assume that the national polices for perinatal care in all country, according to a plan for the regionalization (since 1990), increased the probability of early viability, and consequently, increased the recording of extremely preterm live births since 1990. Due to a lack of link with the register of stillbirth, we cannot value this possibility.

Similarly, the misclassifications and missing values should have been progressively able to integrate the group of preterm births, but their number always remained low and relatively stable, and most of the births with missing values for GA had a BW corresponding to term births. The births with missing values for weight had more preterm deliveries, but this category of birth has diminished markedly since 2001 and could not contribute to recent trends.

The role of other more widespread factors, such as the changes in maternal age signalled in demographic reports [21], remains to be determined, especially the teenaged and older mothers. Studies in Chile report an increase in both groups during the observed period [22] and evidences show that these mothers are at high risk of give birth a child with very preterm birth and low birth weight [23].

Advanced maternal age may be a risk factor, both for the rates of multiple pregnancies but also in terms of greater recourse to treatment for infertility. It has been observed in France that from a quarter to a third of multiple births are related to increase of maternal age, and more than 30% to treatment for infertility [24].

Nonetheless, the increase in multiple births does not appear to be linked to procedures of fertilisation or assisted reproduction, for the elevated cost of these procedures makes them still quite rare. According to the reports of the Latin American Network for the assisted reproduction (REDLARA) only 480 births of this type was born in 2008, 23% of them twin births [25].

### The possible influence of LMP and USN as methods to estimate GA

It is well known that the choice of method for estimating gestational age can influence perinatal outcomes [26]. This should not be surprising: the methods rely on different parameters. LMP measures the duration of gestation while USNF is based on fetal anthropometric measurements [27].

LMP is greatly affected by the individual characteristics of mother and fetus, and the gestational age tends to be greater, while ultrasound classifications consistently skew to younger ages, and tend to predict shorter pregnancies.

As for the effect of these two methods on perinatal outcomes, divers studies show that LMP is associated with a higher incidence of adverse outcomes, including preterm, postterm, and growth fetal restriction [26-29]. Ultrasound estimate also appear to correlate with a greater incidence of premature births than LMP, decreased birth weight as well as a clear reduction of exceeding 41 weeks [29-31].

Ultrasound also may diagnose fetuses smaller than the mean or having growth restriction as having less gestational age. Conversely, fetus determined to be oversized for its gestational age may be classified to a more advanced gestational age [27,32].

In sum, it appears that the use of one single method (US or LMP) has strengths and limitations. We could expect that we can obtain better estimations if the two methods are considered and particularly if there is concordance between their results [26,30].

In Chile, the studies that have assessed the fetal ultrasound program for the first trimester observed good concordance between the date of the last menstrual period and the ultrasound date, and this concordance has remained relatively stable over time (61.9% in 1994 and 65.6% in 2001) [15]. Other studies have confirmed these findings (weighted Kappa: 0.64) [33].

In our study it seems that the choice of the one or the other method did not change the increasing trends in the rate of preterm.

On the other hand, the increased use of ultrasound estimates could have reduced the incidence of errors and missing values as well as in the values exceeding 41 weeks [34,35].

### The decrease in births between 37 and 41 weeks, with a large reduction in births after 41 weeks, may be related to increased medical intervention during pregnancy

Since the 1990s, nationwide clinical guidelines based on high risk approach have been implemented to improve maternal and perinatal health [36]. The large reduction in births after 41 weeks might thus be associated, on the one hand, with first-trimester fetal ultrasonography, and on the other hand, with the termination of at-risk pregnancies.

At the same time, between 1990 and 2000, prenatal care coverage rose from 85% to 91.4%, and the percentage of women who began prenatal care before 20 weeks of gestation rose from 74% in 1994 to 86% in 2000. All these changes probably increased the opportunities for screening for disorders and preventing complications.

In this context, the observed trends may also express medical practices that were more active in the face of maternal or fetal risks and led to more frequent recourse to caesarean birth or induction of labour.

This is the case for the deliveries that, according to national guidelines, are induced from week 41 to diminish fetal risk; this guideline may also help to explain the very low number of postterm infants, as observed elsewhere, as well as the stability of birth weights exceeding 4000 g [35].

Thus, the underlying reason for these preterm birth trends must be considered in future research that must distinguish between spontaneous preterm deliveries and those considered medically indicated.

Observations in 13 European countries show that the countries with the highest rates of induction of labour also have the lowest rates of postterm births [35]. Similarly, researches in North America [37] and South America [38,39] show that excess rates of caesarean deliveries and induction of labour are important contributors to the increase in preterm birth and to the reduction of birth weight.

Several authors have shown that even when taking into account the role of other factors including maternal age, plurality [40,41] or the method for calculating gestational age [34], the effect of the obstetric interventions is clearly of major importance.

According to a trend study conducted from the perinatal information system for the countries of Latin America and the Caribbean (SIP), 40% of preterm births were associated with medical interventions [42]. Deliveries involving induction of labour or elective caesareans have increased over the past 20 years from 10% in 1985 to 18.5% in 2005, accompanied by an increase in preterm and very preterm births. The countries most involved in this increase were Argentina, Brazil and Chile [36].

The caesarean rates are especially high in Chile [43]; during the observed period the national caesarean rate was reported to be 40%: it fluctuates around 30% in public hospitals, and around 50% in private hospitals, exceeding 60% in some facilities [43,44]. The effect of obstetric interventions on mothers and babies has been evaluated [39,43], and studies have shown, for example, that the decline in the category of weight greater than 4000 g is due to deliveries induced before completion of the cycle of major weight gain that occurs after week 37 and can reach 600 g [45].

The impact of these wide-scale medical interventions on mothers is both visible and cumulative. Accordingly, a woman who has already had a caesarean delivery in Chile has a risk of a second caesarean delivery 22 times higher than a primipara [46].

### From a comparative point of view, these trends are consistent with those described throughout both North and South America

In America, the largest increase in preterm birth rates from high-income countries has been reported in the United States. Increase is also important in some medium-income countries, including Brazil. The prevalence rate of PTB in both countries is around 12% [4].

This research shows that in Chile, the overall rate of preterm births and low birth weight may be considered relatively low; but there are specific trends to be considered, such as the increase of newborns with less than 32 weeks of GA. This population is at high risk of morbidity and sequelae; might require specialized management and furthermore could have a significant impact on the public health of the country.

Changes such as those observed in these two perinatal outcomes over a considerable period of time could provide information that can be used in the short or long term in several domains of public health as follow up care, financial planning etc. Even more when these current trends could be prolonged in the absence of other targeted prevention policies.

## Conclusions

From an exhaustiveness register of live births, we have observed in the space of 18 years that the distribution of GA and BW in live births has changed significantly, both for singleton and multiple births.

Three trends resulting from these changes deserve special attention, because of their perinatal health impact. They are: the significant increase in the preterm birth rate, especially in the group of new born with less than 32 weeks of gestational age, the increase in the prevalence of very LBW, and finally, the sustained increase in multiple births.

Although this descriptive study cannot prove the involvement of other factors according to type of birth, trends in preterm birth and LBW were measured with more specificity than previously and the main outcomes raise other challenges for future researches, such as the assessment of obstetrical and perinatal practices and the need to obtain a better profile of maternal population.

This epidemiological information allows us to better define the problems we face and might thus be useful to decision-making bodies for attributing priorities. Equally important is the establishment of a periodic surveillance system for preterm delivery and low BW, both overall and by category, to improve the targeting of public policies related to these indicators.

## Competing interests

The authors declare they have no competing interest in the performance or publication of this research.

## Authors’ contributions

PL Conceived of the study, made possible the acquisition of data, made analysis and interpretation of data. GB Made substantial contributions to conception and design of this study. Has been involved in drafting the manuscript and revised it giving a critical and important intellectual content. Has revised and approved the final version submitted to publish.

## Pre-publication history

The pre-publication history for this paper can be accessed here:

http://www.biomedcentral.com/1471-2393/12/121/prepub
